# The Properties of Poly(ester amide)s Based on Dimethyl 2,5-Furanedicarboxylate as a Function of Methylene Sequence Length in Polymer Backbone

**DOI:** 10.3390/polym14112295

**Published:** 2022-06-05

**Authors:** Konrad Walkowiak, Izabela Irska, Agata Zubkiewicz, Jerzy Dryzek, Sandra Paszkiewicz

**Affiliations:** 1 Department of Materials Technologies, Faculty of Mechanical Engineering and Mechatronics, West Pomeranian University of Technology, PL 70310 Szczecin, Poland; izabela.irska@zut.edu.pl (I.I.); sandra.paszkiewicz@zut.edu.pl (S.P.); 2 Department of Physics, Faculty of Mechanical Engineering and Mechatronics, West Pomeranian University of Technology, PL 70311 Szczecin, Poland; azubkiewicz@zut.edu.pl; 3 Institute of Nuclear Physics Polish Academy of Sciences, PL 31342 Krakow, Poland; jerzy.dryzek@ifj.edu.pl

**Keywords:** poly(ester amide)s, a number of methylene groups, biopolymers, 2,5-furandicarboxylic acid, thermal properties, mechanical performance, PALS

## Abstract

A series of poly(ester amide)s based on dimethyl furan 2,5-dicarboxylate (DMFDC), 1,3-propanediol (PDO), 1,6-hexylene glycol (HDO), and 1,3-diaminopropane (DAP) were synthesized via two-step melt polycondensation. The phase transition temperatures and structure of the polymers were studied by differential scanning calorimetry (DSC). The positron annihilation lifetime spectroscopy (PALS) measurement was carried out to investigate the free volume. In addition, the mechanical properties of two series of poly(ester amide)s were analyzed. The increase in the number of methylene groups in the polymer backbone resulted in a decrease in the values of the transition temperatures. Depending on the number of methylene groups and the content of the poly(propylene furanamide) (PPAF), both semi-crystalline and amorphous copolymers were obtained. The free volume value increased with a greater number of methylene groups in the polymer backbone. Moreover, with a lower number of methylene groups, the value of the Young modulus and stress at break increased.

## 1. Introduction

Most polymers are based on petrochemicals, but the increasing environmental awareness and dwindling fossil fuel resources have made it clear that this needs to be changed. Consequently, there is a growing interest in the use of renewable raw materials for fuels, chemicals, and polymer materials [1]. In 2004, the US Department of Energy recognized 2,5-furanedicarboxylic acid (FDCA) as one of the twelve most important renewable materials [2]. The FDCA and its derivatives find application in the synthesis of bio-based polyurethanes, polyamides, and especially polyesters. The similar structure of FDCA to terephthalic acid (TPA) popularized the synthesis of materials such as poly(ethylene 2,5-furanoate) (PEF) and poly(propylene furanoate) (PPF), which are more environmentally friendly equivalents of poly(ethylene terephthalate) (PET) and poly(propylene terephthalate) (PPT) [1,3,4]. Moreover, due to the heteroatom in the structure of the furan ring, polyesters based on FDCA exhibit excellent gas barrier properties [5,6]. For example, PEF has 11 times better barrier properties to O_2_, and 19 times better to CO_2_ than PET [7,8,9]. As a result, it can be used in the production of films, bottles, and fibers [10].

In order to increase the applicability of furan-based polymers, attention has been paid to poly(ester amide)s (PEA)s. This polymeric class has excellent mechanical and thermal properties, thanks to intermolecular hydrogen bonds that increase interactions between the polymer chains [1]. However, obtaining polyamides and PEA from FDCA has proved to be quite a challenge [11]. It is due to the side reactions, including decarboxylation occurring around 200 °C and the N-methylation of FDCA, that occur [12], whereby the synthesized PEAs have a low molecular weight and are amorphous or have a low degree of crystallinity [13]. Therefore, in the last few years, several attempts to synthesize polyamides and PEAs based on FDCA and its derivatives have been described (e.g., solution polymerization [14], enzymatic polymerization [12,15,16], direct polycondensation [17], and solid-state polymerization [13,18]). One of the strategies involves enzymatic polymerization, which uses an immobilized form of Candida Antarctica lipase b, which serves as a catalyst for polycondensation between dimethyl furan 2,5-dicarboxylate DMFDC and aliphatic diamines [15,19]. This reaction takes place under mild conditions, which solves the problem of decarboxylation and the N-methylation of FDCA. However, materials obtained with the use of this strategy were amorphous or characterized by slow crystallization unless treated with solvents. Kluge et al. [20] synthesized semicrystalline PEAs using symmetric amido diol, 1,10-decanediol, and DMFDC via melt polycondensation. The addition of amide groups caused an increase in the degree of crystallinity and improved the thermal and mechanical properties of the material. The crystallization of PEA can occur with a sufficiently high molecular weight and tight packing of molecules, and the formation of hydrogen bonds between the amide protons and the oxygen atom in the furan ring may appear. Moreover, semicrystalline PEA based on FDCA can only be obtained if there is enough space between the amide group and the furan ring [21]. Increasing the number of methylene groups in the aliphatic diols may provide enough space and makes it easier for PEA to crystalize. However, the parity of the number of methyl groups has a significant effect on the crystallization and phase transition temperatures [22]. Polyesters with an even number of methylene groups have a higher value of phase transition temperatures and crystallize more easily than polyesters with an odd number of methylene groups [22], and this effect should also be observed for PEAs.

Therefore, we have compared two series of PEAs, i.e., with an odd number of the methylene group poly(trimethylene 2,5-furandicarboxylate)-co-poly(propylene furanamide) (PTF-co-PPAF) and with an even number of the methylene group poly(hexamethylene 2,5-furandicarboxylate)-co-poly(propylene furanamide) (PHF-co-PPAF). The influence of various aliphatic diols on the selected properties of materials was investigated, in particular the thermal behavior, free volume changes, and mechanical performance. The details about the molecular structure and physico-chemical properties for these particular series of PEAs were already published in [11,21].

## 2. Materials and Methods

### 2.1. Synthesis of Copoly(amide-esters)

**Figure 1:**
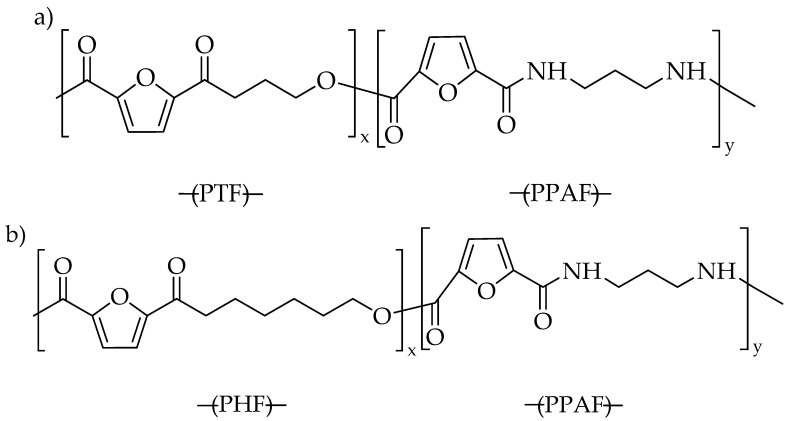
Chemical structure of: PTF-co-PPAF (**a**); PHF-co-PPAF (**b**).

The series of poly(trimethylene 2,5-furanoate)-co-poly(propylene furanamide) (PTF-co-PPAF) and of poly(hexylene 2,5-furanoate)-co-poly(propylene furanamide) (PTF-co-PPAF) copolymers were obtained from dimethyl furan 2,5-dicarboxylate (DMFDC, 99%, Henan Coreychem Co., Ltd., Zhengzhou, China), 1,3-propylene glycol (bio-PDO, DuPont Tate & Lyle BioProducts, Loudon, OH, USA), 1,6-hexylene glycol (HDO, Rennovia Inc., Santa Clara, CA, USA), and 1,3-diaminopropane (DAP, >99%, Sigma Aldrich, Steinheim am Albuch, Baden-Württemberg, Germany). Materials were synthesized via two-step melt polycondensation in a 1 dm^3^ high-pressure reactor (Autoclave Engineers, Erie, PA, USA). In the first step, transesterification between DMFDC, bio-PDO, and DAP in the presence of titanium (IV) butoxide (Ti(OBu)_4_, Fluka^®^ Analytical) as a catalyst and Irganox 1010 (Ciba-Geigy, Switzerland) as an antioxidant was carried out for PTF-co-PPAF. In turn, to obtain PHF-co-PPAF, bio-PDO was replaced with HDO. Transesterification was performed at the range of temperature of 160–190 °C, and the end of the first stage was signaled by distillation of 90% of the theoretical value of the by-product. The second portion of the catalyst (Ti(OBu)_4_) was added before the second step. During polycondensation, the temperature was gradually increased to 220 °C for PTF-co-PPAF and 240 °C for PHF-co-PPAF, and pressure was reduced below 20 Pa for both series. The second stage was finished when the proper value of the melt viscosity was acquired, which was evaluated by observation of a stirring torque change. The materials were then pressed from the reactor into the water bath. The extruded materials were granulated in a laboratory mill and injected into a dumbbell-shaped sample, type A3, using a Boy 15 (Dr BOY GmbH & Co., Neutstadt, Germany) for a tensile test. The injection parameters are summarized in Table 1. The chemical structure of the copolymers is presented in Figure 1.

### 2.2. Characterization Methods

The chemical structure of the obtained materials was studied by ^1^H NMR. Before the experiment, samples were subjected to extraction for 24 h. ^1^H NMR spectroscopy was performed on a Bruker spectrometer (Karlsruhe, Germany) at 400 MHz using chloroform-d CDCl_3_ at a concentration of 10 mg/mL with a few drops of trifluoroacetic acid (CF_3_COOH) to dissolve the samples. Tetramethylsilane (TMS) was used as a reference for the internal chemical shift. The mole fractions of the PPAF segments were calculated from the determined integral intensities of the characteristic peaks, according to Equation (1):

(1) $$W_{\mathrm{PTF}-\mathrm{co}-\mathrm{PPAF}}=\frac{2I_{x}}{2I_{x}+{\;I}_{y}}\times 100{\%\;\mathrm{or}\;W}_{\mathrm{PHF}-\mathrm{co}-\mathrm{PPAF}}=\frac{I_{x}}{I_{x}+2I_{y}}\times 100\%\;$$

where I_x_ and I_y_ are integral signal intensities that correspond to peaks of 3.63 ppm and 2.26 ppm for PTF and PTF-co-PPAF, respectively, and 2.15 ppm and 4.32 for PHF and its based copolymers, respectively.

Differential scanning calorimetry (DSC) was performed using DSC 204 F1 Phoenix (Netzsch, Selb, Germany). A total of 10 mg of each sample were encapsulated in aluminum pans and analyzed in a temperature range from −75 °C to 250 °C with a speed of 10 °C/min. The measurement was carried out under nitrogen flow in a heat/cool/heat cycle. The degree of crystallinity was calculated from Equation (2):

(2) $$x_{c}=\frac{\Delta H_{m}-\Delta H_{x}}{\Delta H_{m}^{0}}$$

where ΔH_m_ and ΔH_x_ are the melt and cold crystallization or crystallization enthalpy, respectively, and $\Delta H_{m}^{0}\;$ is the heat of fusion of the 100% crystalline polyester: for PTF it is 142 J/g [23] and for PHF it is 143 J/g [24].

The thermo-oxidation was investigated by employing TGA 92-16.18 Setaram (Caluire-et-Cuire, France). The measurements were carried out in an oxidizing atmosphere, i.e., dry, synthetic air (N_2_:O_2_ = 80:20 vol.%) at a flow rate of 20 mL/min, with a heating rate of 10 °C/min in the temperature range of 20–700 °C. Measurements were conducted under the PN-EN ISO 11358:2004 standard.

Positron annihilation lifetime spectroscopy (PALS) measurements were carried out on a TechnoAp digital positron lifetime spectrometer with a timing resolution of 190 ps. The *^22^NaCl* between two thin Kapton foils with a thickness of 7 μm was the source of the positron. For the detection of the annihilation and 1.27 MeV gamma photons, two Hamamatsu H3378-50 photomultipliers coupled with a single crystal of BaF2 scintillators from KristalKort were used. Spectras obtained from the measurement were deconvoluted using the LT-Polymer code [25].

The static mechanical properties were investigated using Autograph AG-X plus (Shimadzu, Kyoto, Japan), which is equipped with an optical extensometer, 1 kN Shimadzu load cell, and Trapezium X software. In the beginning, the materials were extended to 1% with a crosshead speed of 1 mm/min. Then, the stress–strain curves for the materials were obtained at a rate of 5 mm/min. The reported values are an average of the five test specimens. The measurements were performed according to PN-EN ISO 527.

## 3. Results

### 3.1. Structure and Composition

**Figure 2:**
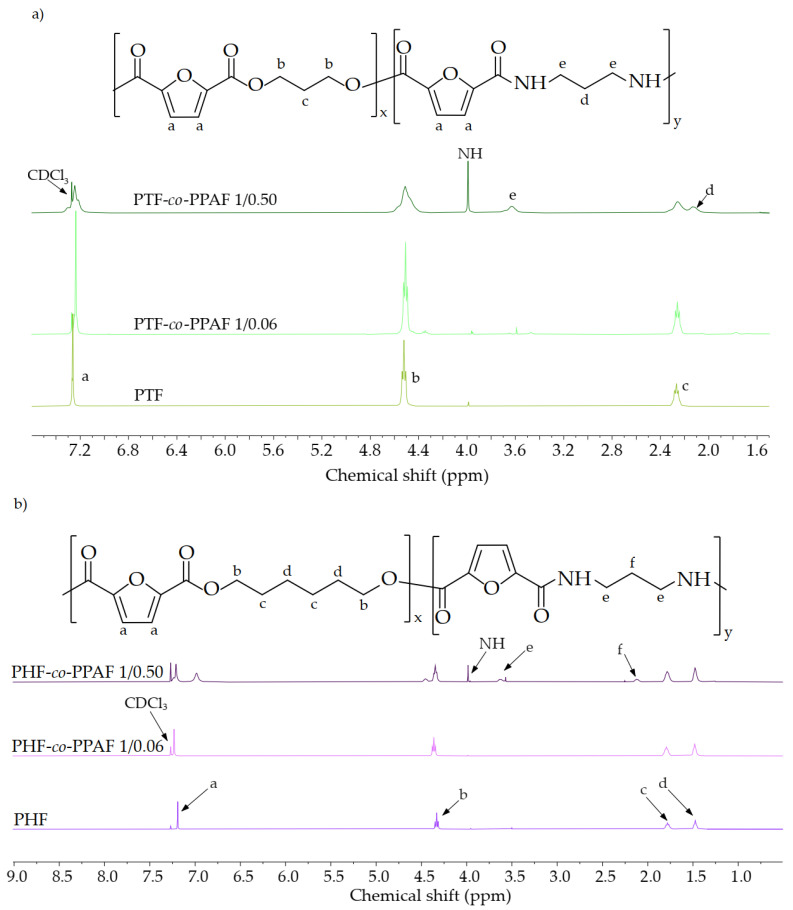
^1^H-NMR spectra of PTF, PTF-co-PPAF 1/0.06, PTF-co-PPAF 1/0.50 (**a**), and PHF, PHF-co-PPAF 1/0.06, PHF-co-PPAF 1/0.50 (**b**).

The chemical structure and composition of the PTF, PHF, and copoly(ester amide)s were confirmed by ^1^H NMR. The ^1^H NMR spectra of the polyesters and copolymers with the lowest and highest content of PPAF are shown in Figure 2. Furthermore, the composition of the obtained materials can be found in Table 2. The signal at 7.24 ppm for PTF and 7.19 for PHF is assigned to the protons of the furan ring. PTF and PHF differed in the signals derived from the methylene group. The peaks occurring at 4.52 ppm and 2.27 ppm are associated with the propylene glycol subunit for PTF, while the peaks at 4.33, 1.79, and 1.48 ppm correspond to the protons of the methylene groups in the hexylene glycol subunit for PHF. However, a weak signal at 2.1 ppm was observed in PHF, which is associated with the central methylene group belonging to 1,3-propanediol. The PTF- or PHF-based copolymers showed signals associated with PPAF, the peak from the protons of the methylene groups adjacent to the nitrogen atom appeared at 3.63 ppm, and the signal from the amide proton was visible at 3.98 ppm. The calculated molar fractions of PPAF were slightly lower than the theoretical values, which could have resulted from the formation of by-products and the lower molecular weight of these materials. For sure, the obtained poly(ester amide)s were found to be random copolymers. This was caused by the almost equal reactivities of the monomers and the random transesterification reaction during the polycondensation process.

### 3.2. Thermal Properties

**Figure 3:**
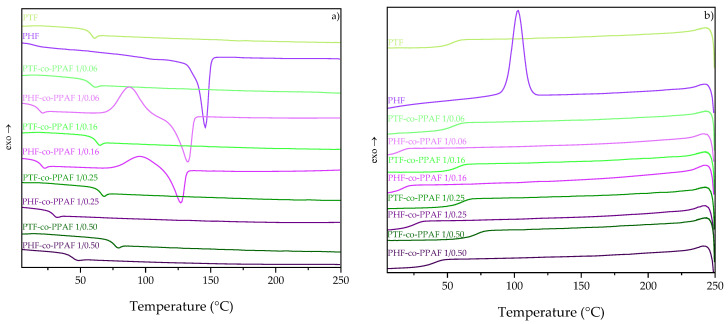
DSC thermograms recorded during second heating (**a**), and cooling (**b**).

In order to investigate the phase transition temperatures and the structure of the synthesized polymers and copolymers, the DSC measurement was performed. The DSC thermograms recorded during the second heating and cooling are shown in Figure 3. Results from the DSC analysis are summarized in Table 3. It is clearly visible that the PHF and PHF-based copolymers exhibited a lower value of glass transition compared to the PTF and PTF-based PEAs since an increase in the alkyl chain length changed the flexibility of the chain due to the internal plasticization of the polymer backbone [3]. Moreover, one can observe that the addition of the PPAF content to the neat polyesters caused an increase in the value of the glass transition temperature in both series. It was due to the hydrogen bonds of the amide groups which increased the stiffness of the molecular chains [3]. Therefore, PTF-co-PPAF 0.50 had the highest glass transition temperature (73.9 °C) compared to the rest of the synthesized polymers. The PTF and PTF-co-PPAF copolymers did not crystallize due to the insufficient space between the amide group and the furan ring and also because polyesters with an odd number of methylene groups do not crystallize as well as polyesters with an even number of methylene groups [22]. Moreover, polyesters with a longer methylene unit sequence length had a better regularity of molecular chains that made it easier for the polyesters to crystallize. Only PHF, PHF-co-PPAF 1/0.06, and PHF-co-PPAF 1/0.16 showed a crystalline structure, although the degree of crystallinity decreased with increasing PPAF content. It was caused by thermodynamic interaction between PHF and PPAF, in which the amorphous PPAF units reduced the thickness of the crystals and became less stable [21,26]. That is why the degree of crystallinity between PHF and PHF-co-PPAF 1/0.16 differed significantly, about 24.7%. Contrary to PHF, which crystallized during cooling, PHF-co-PPAF 1/0.06 and PHF-co-PPAF 1/0.16 were characterized by cold crystallization due to a limited crystallization ability. The cold crystallization temperature increased with a higher molar fraction of PPAF, but the enthalpy of the cold crystallization decreased. Moreover, the melting temperature also decreased with an increasing PPAF content. In addition, the characteristic temperatures of the 5% and the 50% mass loss designated from TGA are shown in Table 3. PTF and its copolymers exhibited higher thermal stability in comparison to PHF and the series of PHF-co-PPAF. This was due to the lower number of methylene groups of PTF and the copolyesters based on it, providing more rigidity to the polymer chains. A more detailed investigation for each series can be found in [11,21].

### 3.3. Positron Annihilation Lifetime Spectroscopy

Positron lifetime spectra were carried out to determine the change of the free volume in the synthesized polymers. They were measured at room temperature and described considering three lifetime components. The first and second components were shorter lifetimes: *τ*_1_ (the annihilation of the para-positronium, p-Ps) and *τ*_2_ (the annihilation of the free position). The third lifetime component *τ*_3_ was attributed to the ortho-positronium (o-Ps) and was commonly related to the annihilation in free volume. The values of *τ*_3_ and *I*_3_ are important because they are used to describe the pick-off process. In this process, the positron in Ps annihilates with an electron other than its associated partner and with the opposite spin during Ps collisions with the surrounding molecules [27]. The pick-off process is represented by a model which uses *τ*_3_ to calculate the free volume radius (*R*) (Equation (3)):

(3) $$\tau_{3}\left[ns\right]=0.5\left[1-\frac{R}{R+\Delta }+\frac{1}{2\pi }\sin\left(2\pi \frac{R}{R+\Delta }\right)\right]$$

where Δ = 0.1656 nm is the parameter determined experimentally for the other molecular solids [27], and *I*_3_ is used for the calculation of free volume fractions (*f_v_*) (Equation (4)):

(4) $$f_{v}=aV_{V}I_{3}$$

where the size of the free volume cavities *V_v_* = 4πR^3^/3 is expressed in Å^3^ and the coefficient a = 0.0018 [28].

The lifetime and intensity values of the components, the free volume radius (*R*), and the free volume fractions (*f_v_*) are summarized in Table 4. The PALS measurement for PHF-co-PPAF was described in our previous article [21]. The *τ*_3_ and the intensity of the component corresponding to the o-PS annihilation (*I*_3_) were in the range of 1.50 to 1.56 ns and 12.6 to 15.6% for PTF and the series of PTF-co-PPAF, respectively. The values of *τ*_3_ did not differ significantly, but the values of *I*_3_ decreased with increasing PPAF concentrations due to the lower density of the free-volume holes. Similar to *τ*_3_, the *R* value for PTF-co-PPAF had a similar value, which indicated no influence of the PPAF content on the free volume radius, Table 4. However, with a higher molar fraction of PPAF in a series of PTF-co-PPAF, the value of the free volume fraction decreased. Before comparing the series of PTF-PPAF and PHF-co-PPAF, it should be noted that PHF, PHF-co-PPAF 1/0.06, and PHF-co-PPAF 1/0.16 have a glass transition temperature below room temperature, at which the measurement was performed. For this reason, these polymers, despite being semicrystalline, did not have a lower value of the free volume radius and free volume fraction in comparison to PHF-co-PPAF 1/0.25 and PHF-co-PPAF 1/0.50. The polymer with the highest value of *τ*_3_ and *I*_3_ was PHF which differed from PTF by about 0.18 ns and 4.8%, respectively. Moreover, PHF and the series of PHF-co-PPAF had more methylene groups than PTF and PTF-co-PPAF, which affected the value of *R* and *f_v_*. The free volume radius value was slightly higher for the series of PHF-co-PPAF, but the value of the free volume fraction was significantly higher in comparison to the series of PTF-co-PPAF. This was due to the reduction of the ester group density with the increase in methylene groups, which reduced the stiffness of the molecular chains and increased the value of *f_v_*.

### 3.4. Mechanical Properties

**Figure 4:**
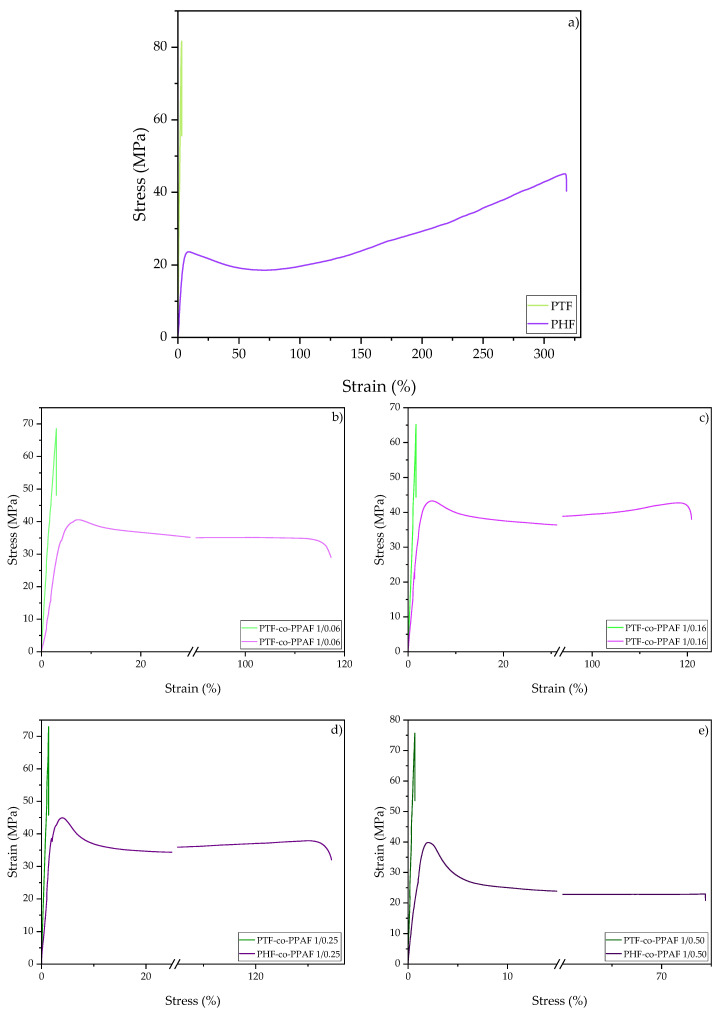
Representative tensile stress–strain curves for: (**a**) PTF, PHF; (**b**) PTF-co-PPAF 1/0.06, PHF-co-PPAF 1/0.06; (**c**) PTF-co-PPAF 1/0.16, PHF-co-PPAF 1/0.16; (**d**) PTF-co-PPAF 1/0.25, PHF-co-PPAF 1/0.25; (**e**) PTF-co-PPAF 1/0.50, PHF-co-PPAF 1/0.50.

The representative stress–strain curves of the synthesized polymers are shown in Figure 4. The values of the tensile modulus (E), tensile strength at yield (σ_y_), elongation at yield (ε_y_), tensile strength at break (σ_b_), and elongation at break (ε_b_) are summarized in Table 5. Unlike PTF and its copolymers, PHF and the series of PHF-co-PPAF exhibited a yield point. The value of E and σ_b_ were higher for the PTF-co-PPAF copolymers due to the higher stiffness caused by a lesser number of methylene groups in comparison to the other synthesized polymers. Moreover, as the PPAF content increased for both series of the copolymers, their E value also increased significantly compared to the neat polyesters. The reason for this was the strong intermolecular hydrogen bonding of the amide groups in the microstructure [29]. However, with an increasing fraction of PPAF, the tensile strength of the copolymers decreased compared to the PTF and PHF; a similar effect was observed by Yang et al. [30]. The PHF and PHF-co-PPAF copolymers had a higher value of elongation at break than PTF and the copolymers based on it. This was due to the higher mobility of their molecular chains caused by a greater number of methylene groups. In addition, only PHF showed a strain-hardening effect caused by the orientation of the macromolecular chains and crystallization during stretching. The samples were conditioned at room temperature, which was higher than the glass transition temperature of PHF, PHF-co-PPAF 1/0.06, and PHF-co-PPAF 1/0.16, before the mechanical property testing. This may have increased the degree of crystallinity resulting from the cold crystallization of these samples. Therefore, an increase in the value of Young’s modulus was observed for PHF-co-PPAF 1/0.06 and PHF-co-PPAF 1/0.16 compared to PHF, while the Young’s modulus value decreased after the PPAF content exceeded 0.16 mol.%. Above this content, the obtained copolymers were fully amorphous, as explained in the DSC section.

## 4. Conclusions

Two series of copoly(ester amide)s synthesized by two-step polycondensation were compared in terms of thermal behavior, free volume changes, and mechanical performance. The morphology and phase transition temperatures were investigated by DSC. PHF and the materials based on PHF containing a lower amount of PPAF, i.e., 0.06 mol.% and 0.16 mol.%, can crystallize. This is a result of the better regularity of the molecular chains and the appropriate distance between the amide group and the furan ring. However, the addition of a higher content of PPAF disturbed the crystallization process and caused an increase in the glass transition temperature. The materials with a greater number of methylene groups had a higher value of the free volume fraction and the volume radius, measured by PALS, in comparison to the materials with a lesser number of methylene groups. The f_v_ decreased with increasing PPAF content. Moreover, the relationship between the mechanical properties and the number of methylene groups was proven. The synthesized materials with a higher number of methylene groups also had a higher elongation at the break value than the polymers with a lower number of methylene groups.

## Figures and Tables

**Table 1:** Injection molding parameters of copolymers.

Series	T_i_ [°C]	P_i_ [MPa]	T_f_ [°C]	P_d_ [Mpa]	t_i_ [s]	t_c_ [s]
PTF-co-PPAF	195	85	30	30	6	15
PHF-co-PPAF	180	40	30	25	5	30

T_i_—temperature of injection; P_i_—injection pressure; T_f_—temperature of form; P_d_—holding down pressure; t_i_—time of injection; t_c_—time of cooling.

**Table 2:** Composition of PTF, PHF, and copoly(ester amide)s based on them.

Material	W_polyester_ (mol.%)	W_PPAF_ (mol.%)	W_PPAF_ ^NMR^ (mol.%)
PTF	100	0	0
PTF-co-PPAF 1/0.06	94	6	4.76
PTF-co-PPAF 1/0.16	84	16	16.67
PTF-co-PPAF 1/0.25	75	25	25.37
PTF-co-PPAF 1/0.50	50	50	45.03
PHF	100	0	0
PHF-co-PPAF 1/0.06	94	6	7.47
PHF-co-PPAF 1/0.16	84	16	15.25
PHF-co-PPAF 1/0.25	75	25	24.24
PHF-co-PPAF 1/0.50	50	50	45.05

W_polyester_—mole fractions of PTF or PHF units; W_PPAF_—mole fractions of PPAF units; W_PPAF_ ^NMR^—mole fraction of PPAF units determined by ^1^H-NMR.

**Table 3:** Thermal properties determined from 2nd heating and cooling thermograms for synthesized polyesters and copolymers.

Sample	T_g_ (°C)	ΔC_p_ (J/g °C)	T_cc_ (°C)	ΔH_cc_ (J/g)	T_c_ (°C)	ΔH_c_ (J/g)	T_m_ (°C)	ΔH_m_ (J/g)	X_c_ (%)	T_5%_ (°C)	T_50%_ (°C)
PTF	56.6	0.40	-	-	-	-	-	-	-	364 ^A^	392 ^A^
PTF-co-PPAF 1/0.06	55.9	0.39	-	-	-	-	-	-	-	366 ^A^	397 ^A^
PTF-co-PPAF 1/0.16	60.1	0.47	-	-	-	-	-	-	-	367 ^A^	400 ^A^
PTF-co-PPAF 1/0.25	63.7	0.41	-	-	-	-	-	-	-	367 ^A^	408 ^A^
PTF-co-PPAF 1/0.50	73.9	0.41	-	-	-	-	-	-	-	360 ^A^	401 ^A^
PHF	15.0	0.12	-	-	103.0	41.9	146.0	36.3	25.4	354 ^B^	388 ^B^
PHF-co-PPAF 1/0.06	17.0	0.41	87.0	32.5	-	-	133.0	34.8	1.6	349 ^B^	386 ^B^
PHF-co-PPAF 1/0.16	19.0	0.33	96.0	18.1	-	-	127.0	19.0	0.7	352 ^B^	388 ^B^
PHF-co-PPAF 1/0.25	28.0	0.32	-	-	-	-	-	-	-	350 ^B^	388 ^B^
PHF-co-PPAF 1/0.50	43.0	0.30	-	-	-	-	-	-	-	334 ^B^	383 ^B^

T_g_—glass transition temperature; ∆C_p_—change of heat capacity; T_cc_, ∆H_cc_—cold crystallization temperature and the corresponding enthalpy of crystallization; T_c_, ∆H_c_—crystallization temperature and the corresponding enthalpy of crystallization; T_m_, ∆H_m_—melting temperature and the corresponding enthalpy of melting, T_5%_—temperature of 5% mass loss, and T_50%_—temperature of 50% mass loss designated at the oxidizing atmosphere for ^A^ the series of PTF-co-PPAF [11] and ^B^ for the series of PHF-co-PPAF [21].

**Table 4:** Results obtained from positron annihilation lifetime spectroscopy.

Sample	*τ*_1_ (ps)	*I*_1_ (%)	*τ*_2_ (ps)	*I*_2_ (%)	*τ*_3_ (ns)	*I*_3_ (%)	*R* (Å)	*V_v_* (Å3)	*f_v_* (%)
PTF	206.5 ± 6.7	34.7 ± 2.0	418.6 ± 10.3	49.7 ± 1.8	1.54 ± 0.01	15.6 ± 0.61	0.24 ± 0.01	0.06 ± 0.01	1.59 ± 0.02
PTF-co-PPAF 1/0.06	207.8 ± 7.3	35.1 ± 1.9	424.5 ± 9.4	50.5 ± 1.8	1.56 ± 0.02	14.5 ± 0.56	0.24 ± 0.01	0.06 ± 0.01	1.51 ± 0.04
PTF-co-PPAF 1/0.16	200.4 ± 5.4	31.5 ± 1.4	406.3 ± 6.2	54.0 ± 1.3	1.51 ± 0.01	14.5 ± 0.41	0.24 ± 0.01	0.05 ± 0.01	1.4 ± 0.04
PTF-co-PPAF 1/0.25	192.4 ± 8.0	31.4 ± 1.9	407.4 ± 9.0	54.9 ± 1.8	1.53 ± 0.02	13.6 ± 0.54	0.24 ± 0.01	0.06 ± 0.01	1.37 ± 0.03
PTF-co-PPAF 1/0.50	188.7 ± 7.6	30.8 ± 1.8	403.7 ± 8.1	56.6 ± 1.7	1.50 ± 0.01	12.6 ± 0.46	0.24 ± 0.01	0.05 ± 0.01	1.21 ± 0.03
PHF	192.3 ± 11.9	27.0 ± 2.7	395.1 ± 11.6	52.6 ± 2.5	1.72 ± 0.01	20.4 ± 1.02	0.26 ± 0.01	0.07 ± 0.01	2.64 ± 0.03
PHF-co-PPAF 1/0.06	184.0 ± 6.0	26.3 ± 1.4	381.5 ± 5.3	54.3 ± 1.2	1.67 ± 0.01	19.4 ± 0.49	0.25 ± 0.01	0.07 ± 0.01	2.34 ± 0.03
PHF-co-PPAF 1/0.16	193.0 ± 9.2	29.1 ± 1.9	389.2 ± 7.1	50.8 ± 1.8	1.68 ± 0.01	20.1 ± 0.71	0.25 ± 0.01	0.07 ± 0.01	2.49 ± 0.03
PHF-co-PPAF 1/0.25	191.3 ± 9.2	31.3 ± 2.3	399.5 ± 11.4	49.9 ± 2.1	1.67 ± 0.01	18.8 ± 0.84	0.25 ± 0.01	0.07 ± 0.01	2.27 ± 0.03
PHF-co-PPAF 1/0.50	186.5 ± 4.9	27.9 ± 1.1	393.4 ± 4.2	53.0 ± 1.0	1.65 ± 0.01	19.1 ± 0.38	0.25 ± 0.01	0.07 ± 0.01	2.28 ± 0.03

*τ*_1,_ *I*_1,_ *τ*_2,_ *I*_2,_ *τ*_3,_ *I*_3,_ positron lifetime and intensity of components, corresponding to the shortest lifetime component (p-Ps), the intermediate component, and the pick-off annihilation of the o-Ps component, respectively; *R*, free volume radius; *V_v_*, size of the free volume cavities; *f_v_*, free volume fractions.

**Table 5:** Mechanical properties of PTF, PHF, and copolymers based on them.

Sample	E (GPa)	σ_y_ (MPa)	ε_y_ (%)	σ_b_ (MPa)	ε_b_ (%)
PTF	2.5 ± 0.4	-	-	81.6 ± 1.6	3.1 ± 0.2
PTF-co-PPAF 1/0.06	2.2 ± 0.2	-	-	68.5 ± 1.0	3.0 ± 0.1
PTF-co-PPAF 1/0.16	2.6 ± 0.2	-	-	64.7 ± 0.6	1.6 ± 0.1
PTF-co-PPAF 1/0.25	3.0 ± 0.2	-	-	72.8 ± 0.7	1.4 ± 0.1
PTF-co-PPAF 1/0.50	3.6 ± 0.1	-	-	75.6 ± 0.9	0.7 ± 0.1
PHF	0.4 ± 0.1	23.6 ± 0.9	8.8 ± 1.0	45.3 ± 2.7	319.2 ± 13.3
PHF-co-PPAF 1/0.06	0.8 ± 0.1	42.4 ± 1.8	6.1 ± 0.9	39.1 ± 4.1	146.3 ± 14.9
PHF-co-PPAF 1/0.16	1.7 ± 0.2	43.3 ± 2.6	5.0 ± 0.4	38.8 ± 3.6	106.6 ± 13.7
PHF-co-PPAF 1/0.25	1.3 ± 0.2	43.7 ± 1.0	2.1 ± 0.2	39.4 ± 2.8	147.1 ± 24.6
PHF-co-PPAF 1/0.50	1.0 ± 0.2	38.4 ± 2.2	2.5 ± 0.7	25.8 ± 3.1	75.3 ± 10.3

E, Young’s modulus; σ_y_, stress at yield; ε_y_, elongation at yield; σ_b_, stress at break; ε_b_, elongation at break.

## Data Availability

The data presented in this study are available on request from the corresponding author. After publication, the data will be kept in the open repositories by means of Mendeley Data and RepOD.
